# Silencing of Aphid Genes by dsRNA Feeding from Plants

**DOI:** 10.1371/journal.pone.0025709

**Published:** 2011-10-05

**Authors:** Marco Pitino, Alexander D. Coleman, Massimo E. Maffei, Christopher J. Ridout, Saskia A. Hogenhout

**Affiliations:** 1 Department of Disease and Stress Biology, The John Innes Centre, Norwich Research Park, Norwich, United Kingdom; 2 Plant Physiology Unit, Department of Plant Biology, Innovation Centre, University of Turin, Turin, Italy; University of Miami, United States of America

## Abstract

**Background:**

RNA interference (RNAi) is a valuable reverse genetics tool to study gene function in various organisms, including hemipteran insects such as aphids. Previous work has shown that RNAi-mediated knockdown of pea aphid (*Acyrthosiphon pisum*) genes can be achieved through direct injection of double-stranded RNA (dsRNA) or small-interfering RNAs (siRNA) into the pea aphid hemolymph or by feeding these insects on artificial diets containing the small RNAs.

**Methodology/Principal Findings:**

In this study, we have developed the plant-mediated RNAi technology for aphids to allow for gene silencing in the aphid natural environment and minimize handling of these insects during experiments. The green peach aphid *M. persicae* was selected because it has a broad plant host range that includes the model plants *Nicotiana benthamiana* and *Arabidopsis thaliana* for which transgenic materials can relatively quickly be generated. We targeted *M. persicae Rack1*, which is predominantly expressed in the gut, and *M. persicae C002* (*MpC002*), which is predominantly expressed in the salivary glands. The aphids were fed on *N. benthamiana* leaf disks transiently producing dsRNA corresponding to these genes and on *A. thaliana* plants stably producing the dsRNAs. *MpC002* and *Rack-1* expression were knocked down by up to 60% on transgenic *N. benthamiana* and *A. thaliana*. Moreover, silenced *M. persicae* produced less progeny consistent with these genes having essential functions.

**Conclusions/Significance:**

Similar levels of gene silencing were achieved in our plant-mediated RNAi approach and published silencing methods for aphids. Furthermore, the *N. benthamiana* leaf disk assay can be developed into a screen to assess which genes are essential for aphid survival on plants. Our results also demonstrate the feasibility of the plant-mediated RNAi approach for aphid control.

## Introduction

RNA interference (RNAi) is a valuable reverse genetics tool to study gene function in various organisms [1]. The process of RNAi was described as ‘post-transcriptional gene silencing’ (PTGS) in plant systems [2] and is a technique well established in numerous eukaryotic systems across kingdoms, e.g. *Caenorhabditis elegans* [3], *Arabidopsis thaliana* [4] and *Drosophila melanogaster* [5].

With the RNAi method, double-stranded RNA (dsRNA) can specifically lower the transcript abundance of a target gene when injected into an organism or introduced into cultured cells [3]. RNAi involves the cleavage of dsRNA precursors into small-interfering RNA (siRNA) of approximately 21 to 23 nucleotides by the enzyme Dicer [6]. These siRNAs are then incorporated into a RNA-induced silencing complex (RISC). Argonaute proteins, the catalytic components of RISC, use the siRNA as a template to recognize and degrade the complementary messenger RNA (mRNA) [6]. RNAi can therefore be exploited to suppress gene expression through highly specific depletion of target transcripts.

Aphids are sap-sucking insects of the order Hemiptera and are important crop pests in terms of direct feeding damage and also transmission of plant viruses [7]. RNAi has been successfully used to investigate gene function in the pea aphid *Acyrthosiphon pisum*, a relatively large aphid that can be injected with dsRNA. Nonetheless, the *A. pisum* host range is predominantly restricted to leguminous species. On the other hand, the green peach aphid *Myzus persicae* can feed on over 40 different plant families [8] and is capable of efficiently transmitting over 100 types of plant viruses [9]. Hence, *M. persicae* is one of the most important aphid pests in agricultural crops. However, RNAi has not previously been documented in this species.

RNAi-mediated gene knockdown can be achieved in aphids through direct injection of dsRNA or small-interfering RNAs (siRNA) into aphid hemolymph [10], [11]. This approach was used to silence *C002*, a gene strongly expressed in the salivary glands of *A. pisum* [10]. Silencing the gene resulted in lethality of the aphids on plants, but not on artificial diet, indicating that C002 has a function in aphid interaction with the plant host [10], [12]. We identified the homologue of *C002* from *M. persicae* and named it *MpC002* [13]. *MpC002* is predominantly expressed in the *M. persicae* salivary glands and transient over-expression of *MpC002* in *Nicotiana benthamiana* improved *M. persicae* fecundity [13]. Microinjection of long dsRNA into *A. pisum* also leads to silencing of genes encoding calreticulin and cathepsin by 30–40% [11]. Calreticulin is a calcium-binding protein that is produced in most aphid tissues, while cathepsin is specifically produced in the pea aphid gut. Thus, gene silencing appears to occur in different aphid tissues [11].

Aphids can be fed on artificial diet, which is sandwiched between thin parafilm membranes. *A. pisum* fed on an artificial diet containing dsRNA corresponding to the aquaporin transcript lead to downregulation by more than 2-fold within 24 hours [14]. Since aquaporin is involved in osmoregulation, this resulted in elevated osmotic pressure in the hemolymph [14]. Feeding of dsRNA targeting vATPase transcripts from an artificial diet achieved a 30% decrease in transcript levels in *A. pisum* and a significant increase in aphid mortality [15].

Both micro-injection and artificial diets are valuable methods for achieving RNAi in aphids. However, dsRNA/siRNA has to be synthesized in both cases and neither treatment is natural for aphids. As RNAi in aphids is indeed feasible, it has the potential to be expanded to include gene knockdown via the delivery of dsRNA from plants (plant-mediated RNAi). This method could allow for studying aphid gene function in the aphid natural habitat and may be useful for control aphid pests in crop production. The plant-mediated RNAi method effectively silences genes of lepidopteran and coleopteran insect species [16], [17] and the brown planthopper, a hemipteran species [18]. However, these insects are larger than aphids and hence consume more plant tissue/sap while feeding. Our goal was to determine if the plant-mediated RNAi approach also silences aphid genes. The green peach aphid *M. persicae* was selected because it has a broad plant host range, including the model plants *N. benthamiana* and *Arabidopsis thaliana* for which transgenic materials can relatively quickly be generated. Furthermore, transgenes can be rapidly expressed in *N. benthamiana* leaves using *Agrobacterium*-mediated transient expression providing the possibility to develop a high-throughput system to assess which genes in the aphid genome are essential for survival of aphids on plant hosts. To test the plant-mediated RNAi approach, we selected two *M. persicae* genes, *MpC002* and *Receptor of Activated Kinase C (Rack-1)* as targets. As discussed above, *MpC002* is predominantly expressed in the aphid salivary gland. In contrast, *Rack-1* is predominantly expressed in the aphid gut.

Rack1 is an intracellular receptor that binds activated protein kinase C (PKC), an enzyme primarily involved in signal transduction cascades [19]. Rack-1 is conserved amongst plants and animals and is an essential multifunctional scaffold protein which physically connects diverse signal transduction components into stable complexes [20]. *Rack-1* binds to integrins [21], has a function in actin organisation [22] and is an integral component of the mammalian circadian clock [23]. Rack-1 from *M. persicae* was identified as a luteovirus-binding protein [19] as it was found to bind in vitro to purified wild type or mutant particles of *Beet Mild Yellows Virus* (BMYV). *Rack-1* is a good candidate for RNAi in aphids as *Rack-1* knockdown has been demonstrated in the nematodes *Caenorhabditis elegans* [24], [25] and *Heterorhabditis bacteriophora*, [26]. Knockdown of *Rack-1* resulted in developmentally defective phenotypes in *C. elegans* including slow growth, embryonic lethality, egg laying defectiveness and sluggishness [24], [25] as well as sterility and abnormal gonad development [26]. *Rack-1* in *Drosophila* functions during oogenesis [27] and is required in early oocyte polarity [28].

We found that the expression of both *MpC002* and *Rack-1* is knocked down when *M. persicae* are fed from transgenic plants that transiently (*N. benthamiana*) and stably (*A. thaliana*) express dsRNA corresponding to *MpC002* and *Rack-1*. Moreover, silenced aphids have reduced progeny production. Thus, plant-mediated RNAi is feasible, and is a useful tool for studying aphid gene function.

## Results

### Expression profiles of RNAi target genes

**Figure 1:**
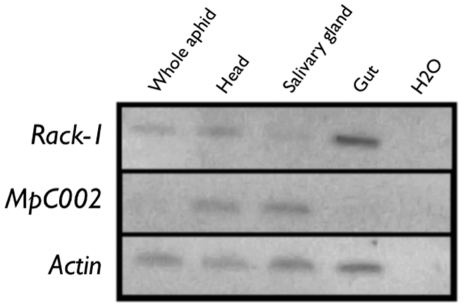
*MpC002* and *Rack-1* are differentially expressed in *M. persicae* tissues. RNA isolated from whole aphids and dissected aphid body parts were used for RT-PCR with specific primers for *Rack-1*, *MpC002* and *Actin*. The latter showed presence of similar RNA concentrations in the aphid samples.

*C002* and *MpC002* are predominantly expressed in the salivary glands of *A. pisum* and *M. persicae* [10], [12], [13], and *Rack-1* in aphid gut tissues [19]. To verify this in our colony of *M. persicae*, RT-PCR was performed on total RNA extracted from different aphid tissues. *MpC002* transcripts were detected in *M. persicae* heads and salivary glands, at relatively low abundance in whole aphids but not in dissected aphid guts (Figure 1). Conversely, *Rack-1* transcripts were found in all aphid body parts and at highest abundance in the gut (Figure 1). These results confirmed previous findings and provided RNAi targets predominantly expressed in the aphid salivary glands and gut.

### Detection of *MpC002* and *Rack-1* siRNAs in *N. benthamiana* leaves

**Figure 2:**
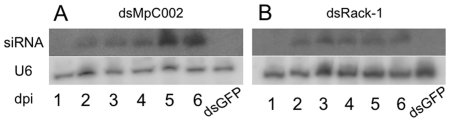
*MpC002* and *Rack-1* dsRNAs are processed into siRNAs (21–23 nt) in agro-infiltrated *N. benthamiana* leaves. *MpC002* and *Rack-1* pJawohl8-RNAi constructs were agro-infiltrated in *N. benthamiana* leaves, which were harvested 1, 2, 3, 4, 5 or 6 days post-inoculation (dpi) for RNA isolation. Total RNA (15–20 µg) was loaded in each lane. Northern blots were hybridized with probes prepared from *MpC002* (**A**) or *Rack-1* (**B**) PCR products. Total RNAs isolated from leaves 6 dpi with GFP pJawohl8-RNAi constructs were included to control for specific hybridization of the *MpC002* and *Rack-1* probes (lanes indicated with dsGFP). To control for equal RNA loading, blots were stripped and then hybridized with an snRNA probe corresponding to U6, which is constitutively produced in plants [45].

First, we investigated if dsRNAs corresponding to *M. persicae MpC002* (dsMpC002) and *Rack-1* (dsRack-1) were produced and processed into siRNAs in *N. benthamiana* leaves. The entire *MpC002* transcript without the region corresponding to the signal peptide (710 bp), a fragment corresponding to the 5′ coding region of the *Rack-1* transcript (309 bp) and a fragment corresponding to the majority of the open reading frame (537 bp) of the green fluorescent protein (GFP) were cloned into the pJawohl8-RNAi plasmid, which expresses the cloned fragments as inverted repeats under control of a double CaMV 35S promoter to produce dsRNAs (I.E. Sommsich, see acknowledgments). Double-stranded GFP (dsGFP) was used as a control for the dsRNA treatments as opposed to empty vector in order to assess whether the presence of dsRNA itself would induce some effect in plant response to aphids. The pJawohl8-RNAi constructs were transiently expressed by *Agrobacterium*-mediated infiltration (agro-infiltration) of *N. benthamiana* leaves. *MpC002* and *Rack-1* siRNAs were observed starting 2 days post agro-infiltration (Figure 2). This indicated that the *MpC002* and *Rack-1* dsRNAs are being processed into 21 to 23 nucleotide siRNAs in *N. benthamiana* leaves. The agro-infiltrated leaves did not show obvious phenotypes such as chlorosis or leaf curling/crinkling upon agro-infiltration of the pJawohl8-RNAi constructs.

### Silencing of *M. persicae MpC002* and *Rack-1* genes by feeding from transgenic *N. benthamiana* leaves

**Figure 3:**
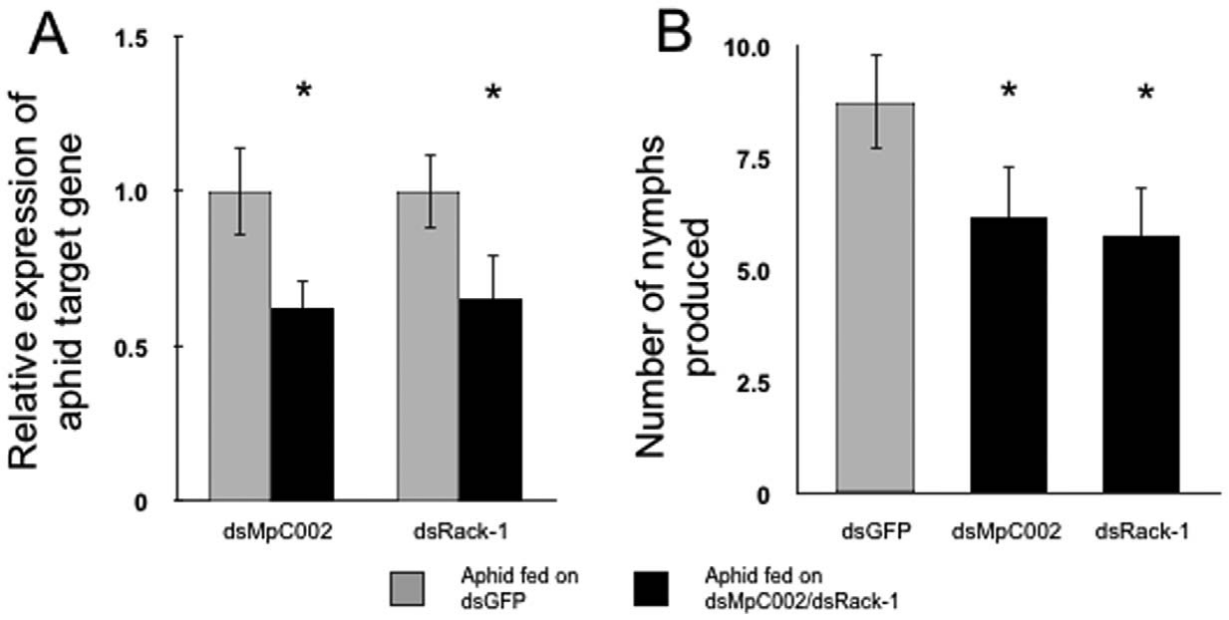
Silencing of *M. persicae MpC002* or *Rack-1* by *N. benthamiana*-mediated RNAi reduces aphid fecundity. (**A**) *MpC002* and *Rack-1* expression is down-regulated in aphids fed on *N. benthamiana* leaves transiently producing *MpC002* and *Rack-1* RNAs. Aphids fed on transgenic *N. benthamiana* leaf discs for 17 days were harvested and analyzed for down-regulation of *MpC002* and *Rack-1* by qRT-PCR. Data shown are means ± standard errors of three biological replicates with n = 3 per replicate. Asterisk indicates significant difference in treatments compared to dsGFP (Student's *t-*test, n = 3, p<0.05) (**B**) *MpC002* and *Rack-1*-silenced *M. persicae* are less fecund. The numbers of nymphs produced by the aphids analyzed for down-regulation of *MpC002* and *Rack-1* in A were counted and compared to the nymphs produced from aphids fed on the dsGFP transgenic *N. benthamiana* leaf discs. Data shown are average number of nymphs produced per adult aphid with means ± standard errors of six biological replicates with n = 4–6 per replicate. Asterisk indicates significant difference in treatments compared to dsGFP (ANOVA, n = 4–6, p<0.05).

Next we investigated if *MpC002* and *Rack-1* are down-regulated in *M. persicae* after feeding on *N. benthamiana* leaves transiently producing the *MpC002* and *Rack-1* RNAs. At one-day post agro-infiltration, 11-mm diameter leaf discs of the infiltrated leaves were placed on top of water agar in wells of 24-well titre plates and exposed to aphids as previously described [13]. Nymphs born on the leaf discs were transferred every 6 days to newly agro-infiltrated leaf discs to ensure continuous exposure of the aphids to the *MpC002* and *Rack-1* RNAs (Figure 2). At 17 days, the adult aphids were collected to assess *MpC002* and *Rack-1* expression levels by quantitative RT-PCR (qRT-PCR). Aphids fed for 17 days on *N. benthamiana* leaf discs infiltrated with dsGFP pJawohl8-RNAi constructs were used as controls. The expression levels of *MpC002* and *Rack-1* were reduced by an average 30–40% compared to the controls (Figure 3A). This downregulation was consistent and highly significant among three biological replicates for *MpC002* (Student's *t-*test, n = 3, p-value = 0.013) and *Rack-1* (Student's *t-*test, n = 3, p-value = 0.012).

### Silencing of aphid *MpC002* and *Rack-1* on stable transgenic *Arabidopsis* lines

**Figure 4:**
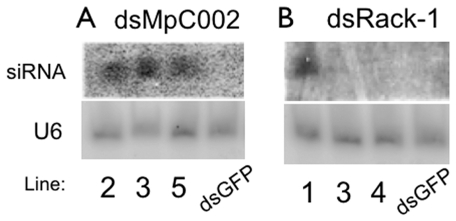
*MpC002* and *Rack-1* dsRNAs are processed into siRNAs (21–23 nt) in transgenic *A. thaliana* lines. Total RNA was isolated from two-week old seedlings of F3 homozygous stable dsMpC002 (**A**) and dsRack-1 (**B**) transgenic lines. Total RNA isolated from two-week old seedlings of a F3 homozygous stable dsGFP line was included to control for specific hybridization (lanes indicated with dsGFP). Each lane contains 15–20 µg of total RNA. Northern blots were hybridized with probes prepared from *MpC002* (**A**) or *Rack-1* (**B**) PCR products. To verify equal RNA loading, blots were stripped and then hybridized with an snRNA probe corresponding to U6, which is constitutively produced in plants [45].

We also investigated the downregulation of *M. persicae* genes *MpC002* and *Rack-1* upon feeding on stable transgenic *A. thaliana* plants. The transgenic lines were obtained by floral-dip transformation of Col-0 plants with the *MpC002*, *Rack-1* and GFP pJawohl8-RNAi constructs used in the *N. benthamiana* transient assays. Three independent F3 homozygous dsMpC002 and dsRack-1 transgenic *A. thaliana* were generated. One F3 homozygous dsGFP transgenic *Arabidopsis* line was included as control. All lines contained the transgenes as confirmed by PCR and sequencing. Northern blot analysis of the transgenic *Arabidopsis* lines revealed the presence of siRNA for *MpC002* and *Rack-1* (Figure 4). The siRNAs corresponding to *M. persicae MpC002* were equally abundant in the three independent transgenic lines (Figure 4A), while the siRNAs corresponding to *Rack-1* were abundant in line 1, less abundant in line 3 and not detected in line 4 (Figure 4B).

**Figure 5:**
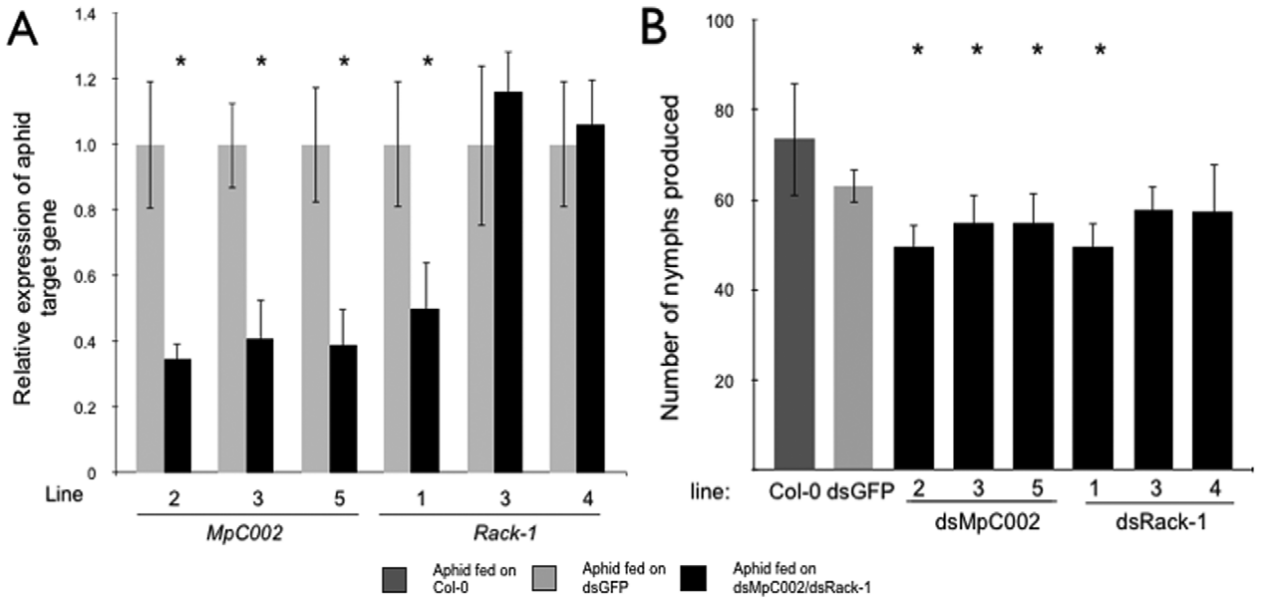
Silencing of *M. persicae MpC002* or *Rack-1* by Arabidopsis-mediated RNAi reduces aphid fecundity. (**A**) *MpC002* and *Rack-1* expression is down-regulated in aphids fed on transgenic Arabidopsis producing *MpC002* and *Rack-1* RNAs. Aphids fed on dsMpC002 or dsRack-1 producing *Arabidopsis* for 16 days were harvested and analyzed for downregulation of *MpC002* and *Rack-1* by qRT-PCR. Data shown are means ± standard errors of three biological replicates with n = 3 per replicate. Asterisk indicates significant difference in treatments compared to dsGFP (Student's *t-*test, n = 3, p<0.05) (**B**) *MpC002* and *Rack-1*-silenced *M. persicae* are less fecund. The numbers of nymphs produced by the aphids analyzed for downregulation of *MpC002* and *Rack-1* in A were counted and compared to the nymphs produced from aphids fed on Col-0. Data shown are total number of nymphs produced on each line with means ± standard errors of three biological replicates with n = 4 per replicate. Asterisk indicates significant difference in treatments compared to dsGFP (GLM, n = 4, p<0.05).

To investigate down-regulation of *M. persicae MpC002* and *Rack-1* on the stable transgenic lines, nymphs born on the transgenic plants were kept on these plants for 16 days at which time the adult aphids were collected for RNA extraction and qRT-PCRs. The aphids reared on three independent dsMpC002 lines showed an approximate 60% decrease in *MpC002* expression compared to aphids reared on dsGFP (Figure 5A). Furthermore, down-regulation of *Rack-1* by approximately 50% was demonstrated for aphids reared on dsRack-1 line 1 compared to dsGFP but not for aphids fed on dsRack-1 lines 3 and 4 (Figure 5A). *MpC002* down-regulation on the three independent lines was consistent in three replicates (Student's *t-*test, n = 3, p<0.05). *Rack-1* was also consistently down-regulated on dsRack-1 line 1 among three replicates (Student's *t-*test, n = 3, p = 0.023), while *Rack-1* was not significantly down-regulated on dsRack-1 lines 3 and 4 (Student's t-test, n = 3, p>0.05). These results are in agreement with the dsMpC002 and dsRack-1 expression levels in the transgenic lines in which the expression of the aphid *Rack-1* gene was not down-regulated on transgenic lines that have low levels of siRNAs corresponding to *Rack-1* (Figure 4B).

### Silencing of *MpC002* and *Rack-1* reduces *M. persicae* fecundity

It was previously shown that silencing of *C002* by injection of dsRNAs in the pea aphid increased the lethality of these aphids on plants [10], [12]. Hence, we assessed if silencing of *MpC002* also affected survival of *M. persicae* feeding directly on *N. benthamiana* and *A. thaliana*. Nymphs exposed to the *N. benthamiana* leaf discs for 17 days became adults and started to produce their own nymphs after approximately 10 days. The overall survival of the aphids and the production of nymphs on leaf discs transiently producing dsMpC002 were not affected compared to aphids on leaf discs producing dsGFP (Figure S1A). However, the nymph production by these aphids was significantly lower in six biological replicates (ANOVA, n = 4–6, p<0.05) (Figure 3B). Similarly, on transgenic *Arabidopsis* plants the *MpC002*-silenced aphids survived equally well, but produced less nymphs in three biological replicates (GLM, n = 4, p<0.05) (Figure S1B, Figure 5B).

Survival and nymph production were also investigated for the *Rack-1*-silenced aphids. *Rack-1*-silenced aphids survived equally well (Figure S1A), but produced fewer nymphs on *N. benthamiana* leaf discs (ANOVA, n = 4–6, p<0.05) (Figure 3B). Similarly, nymph production was reduced on *Rack-1*-silenced aphids feeding on dsRack-1 transgenic *Arabidopsis* line 1 (GLM, n = 4, p<0.05), while survival was not affected (Figure S1B). *M. persicae* fecundity was not reduced on dsRack-1 transgenic *Arabidopsis* lines 3 and 4 (Figure 5B) which is consistent with no significant down-regulation of *Rack-1* in aphids on these lines (Figure 5A).

## Discussion

We have shown that it is possible to down-regulate *M. persicae* gene expression by feeding the aphids dsRNA from plants. As far as we are aware, this is the first example of RNAi in an aphid system from direct plant feeding. We also show that RNAi is possible in *M. persicae*, as RNAi was shown previously in *A. pisum* only.

We measured a 30–60% decrease in gene expression, similar to that observed in microinjection and artificial feeding of small RNAs to aphids. The reduction is also similar to that measured in other insects such as *Schistocerca americana* (injection) [29] and *Rhodnius prolixus* (injection and ingestion) [30] but overall lower than the levels found in *Spodoptera litura* (injection) [31] or in *Drosophila melanogaster* (injection) [32]. Our method allows the study of gene function during interactions of aphids with plants, which is not possible by feeding of dsRNA and siRNA from diets [14], [15].

Previous studies have demonstrated the silencing signal to be mobile in plants [33], where expressed small RNAs to move within the phloem to where aphids feed. The CaMV 35S promoter enables constitutive expression of dsRNA in transgenic plants tissue, including the leaf phloem [34]. The CaMV 35S promoter also allows for transient expression and movement of dsRNAs in *N. benthamiana* phloem [35]. Our results demonstrate that siRNAs can travel from the plant phloem through the aphid stylet and reach the aphid intestinal tissues triggering the silencing of aphid target genes. Given that *MpC002* expression is knocked down by up to 60% and is predominantly expressed in the salivary glands, the silencing signal appears to spread through the aphid. This is consistent with the finding that small RNA pathways that are highly conserved in animals are also present in aphids [36], [37], [38]

Knockdown of *Rack-1* and *MpC002* reduced aphid fecundity (Figure 3B, Figure 5B) but not survival (Figure S1). This contrasts with the results obtained by dsRNA injection of *A. pisum* in which survival was reduced by silencing *C002*. It is possible that the lower *A. pisum* survival is caused by faster downregulation of the target gene as a result of the sudden higher presence of the injected dsRNA in the hemolymph. Alternatively, stress caused by the injection could exacerbate the negative impact of *C002* downregulation. *M. persicae* are smaller than pea aphids and hence more difficult to inject without affecting aphid survival rates. Delivery by plant feeding therefore provides a gentle, natural method for studying gene function that is less likely to have indirect effects on aphid behaviour. Our method is therefore suited to investigating the effects of gene silencing on aphid/plant interactions, and for virus-transmission studies.

*M. persicae* produces more progeny on *N. benthamiana* leaves that transiently express *MpC002* [13]. Thus, the presence of more (*in planta* overexpression) and less (RNAi in aphids) MpC002 leads to, respectively, increased and reduced *M. persicae* performance on plants. In addition, silencing of *A. pisum C002* decreases survival of this aphid on plants but not on diet and the C002 protein was detected in plants upon *A. pisum* feeding [10]. Finally, C002 was found in the saliva proteomes of *M. persicae* [39] and *A. pisum* [40]. Altogether, this indicates that the *C002* genes of both *M. persicae* and *A. pisum* have essential functions in aphid-plant interactions.

Our finding that silencing of *Rack-1* in *M. persicae* leads to decreased progeny production by this aphid is also in agreement with other findings. Indeed, Rack-1 is a scaffold protein that is involved in the regulation of cell proliferation, growth and movement in animals [20], [21], [22]. Silencing of *Rack-1* in two species of nematodes, *C. elegans* and *H. bacteriophora*, reduces growth of these animals [24], [25], [26]. *M. persicae* Rack-1 also interacts with integrins and luteoviruses [19], which invade aphid gut cells [41], suggesting a role in endocytosis processes, such as nutrient/peptide uptake from the gut lumen. Given that *Rack-1* is expressed in multiple tissues of the aphid and particularly in the gut, silencing this gene may affect aphid progeny reproduction indirectly, perhaps by reducing the growth of gut cells leading to decreased nutrient uptake. Alternatively, silencing may directly reduce the growth of embryo cells.

The *M. persicae* genome is being sequenced, but the functions of the majority of aphid genes are still unknown. Moreover, it is not fully understood how aphids modulate host defenses and mediate the transmission of plant viruses. The *N. benthamiana* leaf disc assay can be developed into a functional genomics screen to assess which aphid genes are essential for aphid survival on plants in the absence or presence of specific plant metabolites or synthetic pesticides. It is also possible to further investigate the role of aphid candidate effector proteins in plant infestation [13]. Finally, we can use plant-mediated RNAi to identify aphid proteins involved in the non-persistent and persistent transmission of plant viruses.

## Materials and Methods

### Insect rearing

The aphid lineage used in this study is *Myzus persicae*, lineage of RRes (genotype O) [13]. *M. persicae* were reared on *Nicotiana tabacum* plants for *Nicotiana benthamiana* leaf disc assays and on Chinese cabbage (*Brassica rapa*) for the fecundity assays on *Arabidopsis thaliana*. The insects were maintained in custom-built acrylic cages located in controlled environment conditions at 18°C under 16 hours of light.

### Cloning

Total RNA was extracted using the TRIzol Reagent (Invitrogen, Paisley, UK) and the synthesis of cDNA was performed with poly-T primers using the M-MLV reverse transcriptase system (Promega, Southampton, UK) according to the manufacturer's instructions. *MpC002* and *Rack-1* coding sequences were amplified from *M. persicae* cDNA by PCR with specific primers containing additional attb1 and attb2 linkers (Table S1) for cloning with gateway system (Invitrogen). The *Myzus persicae* EST dataset was mined for the transcript sequences of both target genes [42]. A 710-bp *MpC002* fragment corresponding to the entire mature MpC002 protein without the signal peptide, a 309-bp *Rack-1* fragment starting at nucleotide position +49 ( GGGTTAC) and ending at nucleotide position +358 (CGTCAAA ) of the Rack1 transcript sequence, and a 537-bp GFP fragment starting at nucleotide position +29 ( GAGTGG) and ending at nucleotide position +566 (…TTAGCAG ) of the GFP open reading frame were introduced into pDONR™207 (Invitrogen) plasmid using Gateway BP reaction and transformed into DH5α. Subsequent clones were sequenced to verify correct size and sequence of inserts. Subsequently, the inserts were introduced into the pJawohl8-RNAi binary silencing vector (kindly provided by I.E. Somssich, Max Planck Institute for Plant Breeding Research, Germany) using Gateway LB reaction generating plasmids pJMpC002, pJRack-1 and pJGFP, which were introduced into *A. tumefaciens* strain GV3101 containing pMP90RK plasmid and used for transient assays in *N. benthamiana* leaves and transformation of *A. thaliana*.

### *N. benthamiana* leaf infiltration and leaf disc assays

Single *Agrobacterium* colonies harboring pJMpC002, pJRack-1 or pJGFP were inoculated into Luria Broth (LB) containing 25 mg/l Kanamicin, 25 mg/l Gentamicin, 50 mg/l Rifampicin and 25 mg/l Carbenicillin and grown (28°C at 225 rpm) until an Optical Density (OD_600 nm_) of 0.3 was reached (Eppendorf® BioPhotometer™, Eppendorf, Cambridge, UK). Cultures were resuspended in infiltration medium (10 mM MgCl_2_, 10 mM MES 2-(*N*-morpholino)ethanesulfonic acid, pH 5.6) with 150 µM Acetosyringone to initiate expression. Each construct was infiltrated into the youngest fully expanded leaves of 4–6-week old *N. benthamiana* plants. The plants were grown in a growth chamber with daily temperatures ranging between 22°–25°C under a short day regime. One day after infiltration, leaves were harvested and used in leaf disc assays. The leaf discs were cut from the infiltrated areas using an 11 mm diameter borer and placed in single wells of a 24-well plate on top of a plug consisting of 1 ml solidified 1% distilled water agar (DWA). Four 1^st^ instar nymphs (1–2 days old) reared on *N. tabacum* were places onto the leaf discs for a total of 6 leaves per construct. The wells were individually sealed with mesh and put upside down in controlled environment conditions at temperature 18°C under 16 hours of light. The 24-well plate was replaced with freshly infiltrated (one day post infiltration) leaf discs after 6 and 12 days. Aphid survival by counting was assessed at 6, 12, 14 and 17 days after the day of transfer of aphids to the first 24-well plate and the numbers of nymphs produced by these aphids at 12, 14 and 17 days were also counted. The nymphs were removed after counting. This experiment was repeated 6 times to generate 6 independent biological replicates each containing 6 leaf discs per construct.

### Generation of transgenic plants

The pJMpC002, pJRack-1 or pJGFP constructs were transformed into *A. thaliana* ecotype Col-0 using the floral dip method (Bechtold et al., 1993). Seeds were sown and seedlings were sprayed with phosphinothricin (BASTA) to select for transformants. F2 seeds were germinated on Murashige and Skoog (MS) medium supplemented with 20 µg ml BASTA for selection. Plant ratio of 3∶1 dead/alive (evidence of single insertion) segregation, were taken forward to the F3. Seed from F3 were sown on MS+BASTA and lines with 100% survival ratio (homozygous) were selected. The presence of MpC002/Rack-1/GFP inserts was confirmed by PCR and sequencing. Three independent lines were chosen for dsMpC002/dsRack-1 and one for dsGFP.

### *M. persicae* survival and fecundity assay on *Arabidopsis* transgenic lines

F3 seed were sown and seedlings were transferred to single pots (10 cm diameter) and transferred to an environmental growth room at temperature 18°C day/16°C night under 8 hours of light. Five *M. persicae* adults were confined to single four-week-old *Arabidopsis* lines in sealed experimental cages containing the entire plant. Two days later adults were removed and five nymphs remained on the plants. The number of offspring produced on the 10th, 14th, 16th day of the experiment were counted and removed. This experiment was repeated three times to create data from three independent biological replicates with four plants per line per replicate.

### Northern blot analysis

To assess siRNA accumulation levels by northern blot analyses, *N. benthamiana* leaves were harvested each day for 6 days after agro-infiltration with the pJawohl8-RNAi constructs and whole two-week-old *A. thaliana* F3 transgenic seedlings were used.

Total RNA was extracted from leaves/seedlings using TRIzol reagent (Invitrogen). 15 µg of total RNA was resolved on a 15% polyacrylamide gel (15% acrylamide-bisacrylamide solution 19∶1/7 M urea/20 mM MOPS pH 7.0) and blotted to a Hybond-N membrane (Amersham, Little Chalfont, UK) by a Trans-blot™ (Biorad, Hempstead, UK) semi-dry transfer cell. Cross-linking of RNA was performed by incubating the membrane for two hours using a pH 8.0 solution of 0.2 M 1-Ethyl-3-(3-dimethylaminopropyl)carbodiimide (EDC) (Sigma-Aldrich, Gillingham, UK) and 0.1 M 1-methlyimidazol (Sigma-Aldrich). DNA probes were labeled using Klenow fragment (Ambion, Lingley House, UK) with [α-32P] dCTP to generate highly specific probes. To control for equal loading of RNA amounts, blots were hybridized with a probe to U6 (snRNA 5′-GCTAATCTTCTCTGTATCGTTCC-3′) [43]. MicroRNA marker (NEB, Hitchin, UK) consisting of three synthetic single-stranded RNA oligonucleotides of 17, 21 and 25 residues was loaded in gels and hybridized on blots with corresponding microRNA probe to determine size of siRNA between 21–23 nucleotides. The signals were detected after 3-day exposure to phosphor storage plates (GE Healthcare, Little Chalfont, UK) scanned with a Typhoon™ 9200 scanner (GE Healthcare) and analyzed using ImageQuant™ (GE Healthcare).

### Quantitative real-time PCR analysis

Total RNA was extracted from adult *Myzus persicae* after *A. thaliana* and *N. benthamiana* fecundity assays using TRIzol reagent. DNA contaminations were removed by treating RNA extraction with RNase-free DNase (QIAGEN, West Sussex, UK) and purified with QIAamp columns (QIAGEN). First-strand cDNA was synthesized at 37°C from total RNA using M-MLV (Invitrogen) reverse transcriptase according to the manufacturer's instructions.

Each reaction contained 1 µl of cDNA, 0.5 µl of each specific primers (10 pmol/µl) (Table S1), and 10 µl of 2× SYBR Green Super-mix reagent (Bio-Rad) in a final volume of 20 µl. The following PCR program was used for all PCR reactions: 90°C for 3 m, followed by 40 cycles of 95°C for 30 s, 60°C for 30 s, 72°C for 30 s followed by 10 m at 72°C at the end. Threshold cycle (CT) values were calculated using Bio-Rad CFX Manager™ software (Bio-Rad).

The CT values were normalized for difference in cDNA amount using ßTubulin and L27 CT values [10], [14]. Fold changes were calculated by comparing the normalized transcript levels of *MpC002* and *Rack-1* of *M. persicae* fed on dsMpC002 and dsRack-1 transgenic plants to aphids fed on dsGFP transgenic plants.

### Statistical analyses

All statistical analyses were conducted using GenStat 11 statistical package (VSNi Ltd, Hemel Hempstead, UK) (Table S2, Table S3). Data were checked for approximate normal distribution by visualising residuals. Classical linear regression analysis using a generalized linear model (GLM) with Poisson distributions was applied to analyse the *M. persicae* fecundity data on *A. thaliana* transgenic lines, with “nymphs” as a response variate. The aphid nymph production on 4 plants per treatment was used as independent data points in statistical analyses in which the biological replicate was used as a variable.

*N. benthamiana* leaf disc assay fecundity data were analyzed using an unbalanced one-way ANOVA design with “construct” as the treatment and “repeat” as the block. In the *N. benthamiana* leaf disc assay, aphid fecundity was monitored on individual leaf discs at 6 discs per treatment. Numbers of aphid nymph produced on each leaf disc were used as independent data points in statistical analyses in which the biological replicate was used as a variable. Leaf discs that dried up because of lack of humidity were excluded giving 4–6 leaf discs per construct for each biological replicate. The relative gene expression data were analyzed using 2^−ΔΔC^_T_ method as previously described [44]. The results were analyzed for significant difference with Student's *t-*test. For replication, ‘n = ?’ refers to number of technical replicates used for each variable in each biological replicate i.e. n = 4 *Arabidopsis* plants per line per biological replicate, n = 4–6 *N. benthamiana* leaf discs per construct per biological replicate, n = 3 technical replicates per qRT-PCR biological replicate.

## Supporting Information

Figure S1 **Aphid survival is not affected on** ***dsRack-1*** **and** ***dsMpC002*** **transgenic plants.** (**A**) Aphid survival is not different on dsMpC002, dsRack-1 and dsGFP *N. benthamiana* leaf discs. Data shown are means ± standard errors of aphid survival at 16 days for 6 biological replicates with n = 4–6 per replicate. The relatively low aphid survival on *N. benthamiana* is likely due to transfer of aphids between leaf discs. (**B**) Aphid survival is not different on stable dsMpC002, dsRack-1 and dsGFP transgenic *Arabidopsis* lines for 16 days compared to those fed on dsGFP and Col-0 controls. Data shown are means ± standard errors of aphid survival at 16 days for 3 biological replicates with n = 4 per replicate.
(TIF)

Table S1 **Primer sequences.**
(DOCX)

Table S2 **Statistical analysis data for aphid gene silencing and fecundity experiments on** ***N. benthamiana*** **.**
(DOCX)

Table S3 **Statistical analysis data for aphid gene silencing and fecundity experiments on** ***Arabidopsis*** **.**
(DOCX)
