# Supplementary figures and images for: Silencing of Aphid Genes by dsRNA Feeding from Plants
Source: PLoS One. 2011 Oct 5;6(10):e25709. doi: 10.1371/journal.pone.0025709 (PMC3187792; doi:10.1371/journal.pone.0025709)

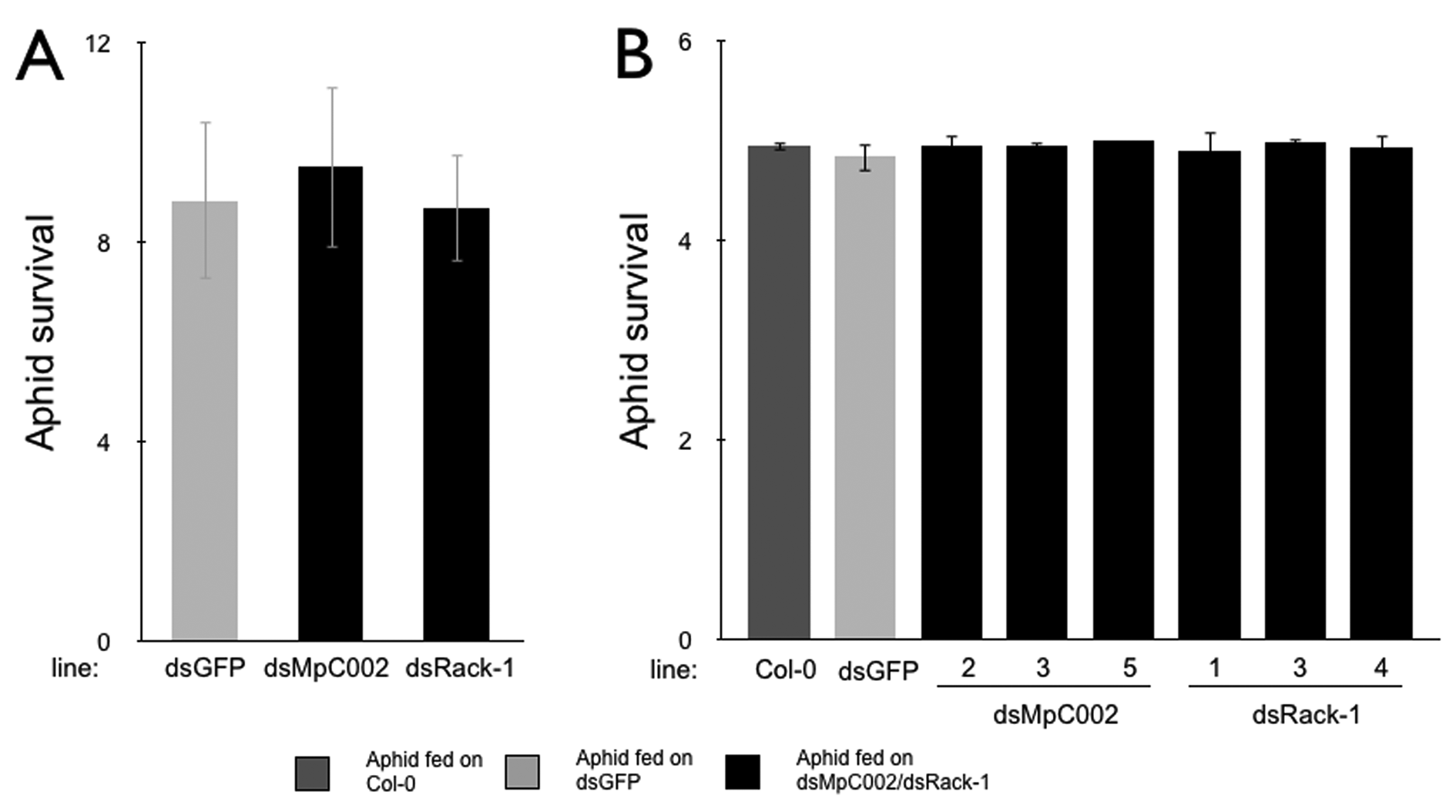

Supplement: Figure S1 — Aphid survival is not affected on dsRack-1 and dsMpC002 transgenic plants. (A) Aphid survival is not different on dsMpC002, dsRack-1 and dsGFP N. benthamiana leaf discs. Data shown are means ± standard errors of aphid survival at 16 days for 6 biological replicates with n = 4–6 per replicate. The relatively low aphid survival on N. benthamiana is likely due to transfer of aphids between leaf discs. (B) Aphid survival is not different on stable dsMpC002, dsRack-1 and dsGFP transgenic Arabidopsis lines for 16 days compared to those fed on dsGFP and Col-0 controls. Data shown are means ± standard errors of aphid survival at 16 days for 3 biological replicates with n = 4 per replicate. (TIF) [file pone.0025709.s001.tif]
