# Supplementary material for: Silencing of Aphid Genes by dsRNA Feeding from Plants
Source: PLoS One. 2011 Oct 5;6(10):e25709. doi: 10.1371/journal.pone.0025709 (PMC3187792; doi:10.1371/journal.pone.0025709)
Supplement: Table S1 — Primer sequences. (DOCX) [file pone.0025709.s002.docx]

**Table S1. Primer sequences.** Primers used in Gateway cloning, qRT-PCR and RT-PCR experiments.

| **Primer name** | **Sequence 5’-3’** |
| --- | --- |
| GFP ATTB1 | AAAAAGCAGGCTGGGAGTGGTCCCAGTTCTTGT |
| GFP ATTB2 | AGAAAGCTGGGTGCTGCTAATTGAACGCTTCC |
| MpC002 ATTB1 | AAAAAGCAGGCTCCATGAAGGTTCAGACTTCCG |
| MpC002 ATTB2 | AGAAAGCTGGGTCTTAAAAATGTCTAAAGAAACGTCC |
| Rack-1 ATTB1 | AAAAAGCAGGCTCCGGGTTACGCAGATCGCCACC |
| Rack-1 ATTB1 | AGAAAGCTGGGTCTGTTTTGACGGTTGTCAGCAGAG |
| ATTB1 ADAPTER | GGGGACAAGTTTGTACAAAAAAGCAGGCT |
| ATTB2 ADAPTER | GGGGACCACTTTGTACAAGAAAGCTGGGT |
| MpC002 F | ACGATGATGAGGGAGGAGTG |
| MpC002 R | GGGTTGCTAAATGCATCGTT |
| L27 F | CCGAAAAGCTGTCATAATGAAGAC |
| L27 R | GGTGAAACCTTGTCTACTGTTACATCTTG |
| Rack-1 F | GGCAAGTGTCTGTCAGTGCT |
| Rack-1 R | ATGCCCATATGCACAAGTCA |
| ßTubulin F | CCATCTAGTGTCGCTGACCA |
| ßTubulin R | GTTCTTGGCGTCGAACATTT |
| Actin F | CGGTTCAAAAACCCAAACCAG |
| Actin R | TGGTGATGATTCCCGTGTTC |
