# Supplementary material for: Silencing of Aphid Genes by dsRNA Feeding from Plants
Source: PLoS One. 2011 Oct 5;6(10):e25709. doi: 10.1371/journal.pone.0025709 (PMC3187792; doi:10.1371/journal.pone.0025709)
Supplement: Table S2 — Statistical analysis data for aphid gene silencing and fecundity experiments on N. benthamiana . (DOCX) [file pone.0025709.s003.docx]

**Table S2. Statistical analysis data for aphid gene silencing and fecundity experiments on *N. benthamiana*.** *Rack-1* and *MpC002* are down-regulated in aphids fed on dsRack-1 or dsMpC002, respectively, compared to dsGFP from three biological replicates (Student’s *t-*test, n=3, p<0.05). Aphids fed on dsRack-1 or dsMpC002 expressing *N. benthamiana* leaf discs for 17 days are significantly less fecund compared to aphids fed on dsGFP from six biological replicates (ANOVA, n=4-6, p<0.05).

|  | **qRT-PCR *N. benthamiana* (P-value)** | **Fecundity on *N. benthamiana* (P-value)** |
| --- | --- | --- |
| **dsRack-1** | Student’s *t*-test p = 0.013 | ANOVA p = 0.001 |
| **dsMpC002** | Student’s *t*-test p = 0.012 | ANOVA p = 0.003 |
