# Supplementary material for: Silencing of Aphid Genes by dsRNA Feeding from Plants
Source: PLoS One. 2011 Oct 5;6(10):e25709. doi: 10.1371/journal.pone.0025709 (PMC3187792; doi:10.1371/journal.pone.0025709)
Supplement: Table S3 — Statistical analysis data for aphid gene silencing and fecundity experiments on Arabidopsis . (DOCX) [file pone.0025709.s004.docx]

**Table S3. Statistical analysis data for aphid gene silencing and fecundity experiments on *Arabidopsis*.** Expression of *Rack-1* and *MpC002* is significantly reduced for aphids fed on *Arabidopsis* dsRack-1 line 1 and dsMpC002 lines 2, 3 and 5 compared to those fed on *Arabidopsis* dsGFP (Student’s *t-*test, n=3, p<0.05). In addition, silenced aphids produced significantly less progeny (GLM, n=4, p<0.05). Aphid gene expression and fecundity were not significantly lower on *Arabidopsis* dsRack-1 lines 3 and 4.

|  | **qRT-PCR *Arabidopsis*** **(P-value)** | **Fecundity on *Arabidopsis*** **(P-value)** |
| --- | --- | --- |
| **dsMpC002 line 2** | Student’s *t*-test p = 0.0001 | GLM p = 0.001 |
| **dsMpC002 line 3** | Student’s *t*-test p = 0.014 | GLM p = 0.034 |
| **dsMpC002 line 5** | Student’s *t*-test p = 0.022 | GLM p = 0.001 |
| **dsRack-1 line1** | Student’s *t*-test p = 0.023 | GLM p = 0.001 |
| **dsRack-1 line 3** | Student’s *t*-test p = 0.36 | GLM p = 0.135 |
| **dsRack-1 lines 4** | Student’s *t*-test p = 0.57 | GLM p = 0.505 |
